# Pleiotropic effects of GDF-15 to regulate nutritional status: perspectives from body composition to nutrition-related disorders

**DOI:** 10.3389/bjbs.2026.16085

**Published:** 2026-07-22

**Authors:** Binbin Peng, Wei Zhao, Ming Kong, Yu Chen, Chao Sun

**Affiliations:** 1 Department of Gastroenterology and Hepatology, Tianjin Medical University General Hospital, Tianjin, China; 2 Fourth Department of Liver Disease, Beijing Youan Hospital, Capital Medical University, Beijing, China; 3 Beijing Municipal Key Laboratory of Liver Failure and Artificial Liver Treatment Research, Beijing, China; 4 Department of Gastroenterology, The First Affiliated Hospital of Xi’an Medical University, Xi’an, Shaanxi, China

**Keywords:** GDF-15, nutrition, body composition, energy metabolism, appetite, cachexia

## Abstract

Growth differentiation factor 15 (GDF-15), a stressful cytokine of the transforming growth factor-β (TGF-β) superfamily, plays pivotal roles in diverse physiological and pathological processes. Most recently, its pleiotropic effects regarding energy metabolism and nutritional modulation are of intense scrutiny. Its physiological functions involve signaling pathways such as GDF-15/glial cell-derived neurotrophic factor family receptor α-like protein (GFRAL)/rearranged during transfection (RET), phosphatidylinositol 3-kinase (PI3K)/Protein kinase B (Akt)/mammalian target of rapamycin (mTOR), nuclear factor kappa B (NF-κB), and reactive oxygen species (ROS). The mechanism of action involves regulation of energy metabolism, inflammation, oxidative stress, and muscle-fat-bone metabolism, and it holds important prognostic value and potential therapeutic significance. Mounting evidence has suggested that GDF-15 levels are significantly increased in the context of cancer cachexia, metabolic syndrome (MetS), and amongst older patients, paralleling multiple indicators of nutrition. Notably, this association appears to be gender- and age-specific, therefore serving as a good biomarker alongside a therapeutic target to mitigate or even reverse disease-related nutritional deficiency and aggravation. However, the precise contribution of GDF-15 to evaluate nutritional status and its mechanistic basis across varying disorders is not fully elucidated. In light of these knowledge gaps, we sought to delve into basic research, clinical information and translational evidence, aiming to analyze the molecular regulatory network of GDF-15 and clarify its clinical implications as a novel diagnostic tool and assessment metric, and in turn provide a theoretical basis for early intervention and personalized treatment. Future research should focus on elucidating GDF-15’s neuroanatomical basis and signaling pathways, validating gender-disparity mechanisms, establishing clinical diagnostic thresholds, optimizing targeted therapeutic strategies, and developing dynamic monitoring approaches, so as to bridge the gap from biomarker discovery to precision intervention.

## Introduction

**FIGURE 1:**
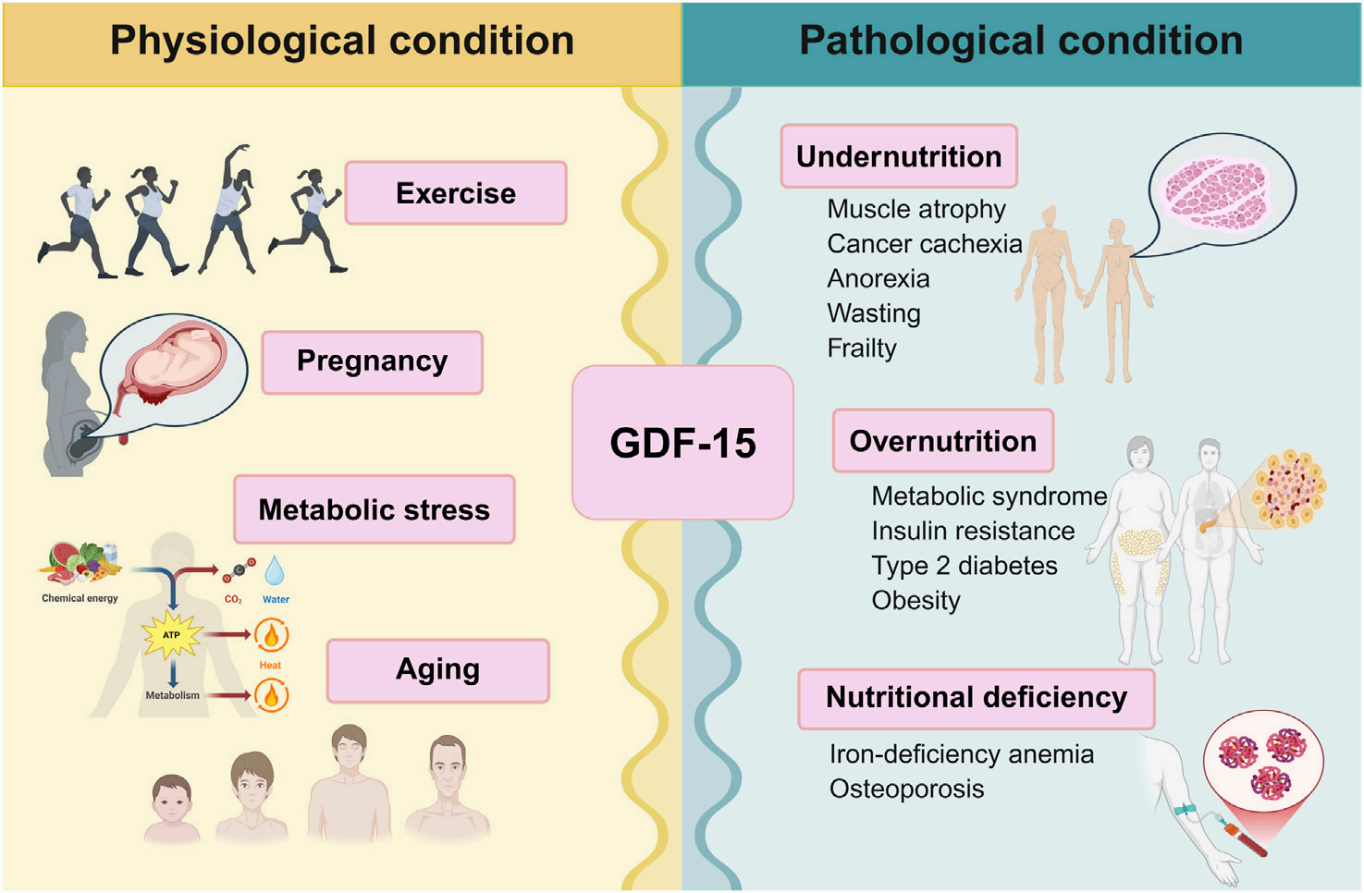
The dual roles of GDF-15 under physiological and pathological conditions. This infographic illustrates the multifaceted implications of growth differentiation factor 15 (GDF-15) in human health. On the left, under physiological conditions, GDF-15 mediates adaptive responses, including metabolic adjustments during exercise, maternal physiological adaptations in pregnancy, metabolic stress responses (e.g., energy homeostasis regulation), and age-related physiological changes. On the right, pathological conditions are characterized by dysregulated GDF-15 signaling, which is linked to adverse outcomes as follows: (1) Undernutrition-related disorders: muscle atrophy, cancer cachexia, anorexia nervosa, wasting syndrome, and frailty; (2) Overnutrition-related disorders: MetS, IR, type 2 diabetes, and obesity; (3) Nutritional deficiency states: iron-deficiency anemia and osteoporosis. This contrast underscores GDF-15’s role as a biomarker that bridges physiological adaptation and pathological dysregulation in nutritional and metabolic health.

Growth differentiation factor 15 (GDF-15) is a member of the transforming growth factor-β (TGF-β) superfamily and has been initially identified as a factor secreted by activated macrophages (i.e., macrophage inhibitory cytokine-1), with the gene located at chromosomal locus 19p13.11 [1]. GDF-15 is represented in multiple forms, including the pro-GDF-15 monomer, pro-GDF-15 dimer, N-terminal pro-fragment of the peptide, and the mature GDF-15 dimer [2]. GDF-15 is widely distributed across a spectrum of organs and tissues with certain tissue predominance: the placenta is the main expression site, followed by the prostate, adipose tissue, liver, skeletal muscle and heart in that order [3]. Its expression is regulated by various factors, such as inflammation, tumors, ionizing radiation, and drugs [4]. More recently, the concept that GDF-15 may induce appetite suppression and weight loss has been well established, although the exact mechanism remains elusive [5]. Until 2017, multiple studies successfully identified glial cell-derived neurotrophic factor family receptor α-like protein (GFRAL) as a specific receptor for GDF-15 to regulate appetite and weight [6–8], exclusively representing the defined receptor for GDF-15. GFRAL, a distant homologue of the glial cell-derived neurotrophic factor family, is expressed in the area postrema (AP) of the hindbrain, a key region involved in regulating nausea, vomiting, and appetite [6]. It plays important roles in neuroprotection, brain development, and energy metabolism. Under physiological conditions, the median level of GDF-15 in human blood fluctuates between 0.4–0.5 ng/mL (as reported by Kralisch S et al.) [9, 10]. Under specific physiological conditions, such as exercise [11, 12], aging [13], and pregnancy [14], its circulating levels increase, but remain lower than those in pathological entities [15]. Across multiple disease conditions like cancer cachexia, cardiovascular diseases, and muscle-fat-bone metabolic disorders, its expression level is reported to be significantly upregulated; meanwhile, it is partially suggestive of the magnitude and prognosis of various diseases [16] (Table 1; Figure 1). From a physiological perspective, GDF-15 exhibits pleiotropic effects. Its core functions cover: (1) Regulation of energy metabolism: GDF-15 reduces food intake through the central appetite-suppressing pathway and affects energy balance in the models of obesity and cachexia [6]; (2) Tissue protection: In patients with acute injuries, GDF-15 promotes tissue homeostasis by impeding the inflammatory burst (e.g., macrophage activation) and strengthening protective molecules (e.g., renal Klotho protein) [29]; (3) Embryonic development: GDF-15’s high expression in the placenta implicates its involvement in the developmental embryonic process [30]; (4) Modulation of iron metabolism: GDF-15 affects erythropoiesis and iron homeostasis by regulating hepcidin [24, 25, 31].

**TABLE 1:** Dual roles and controversies of GDF-15 in body composition and nutrition-related disorders.

Type		GDF-15 beneficial effects	GDF-15 Harmful effects	Contradictions and controversies	References
Body composition	Disease				
Energy metabolism	Cancer Anorexia	Predict disease prognosis Antibodies as therapeutic interventions for cachexia	Promote the occurrence of cachexia Induce tumor immune escape	GDF-15 is derived not only from tumors but also from other organs, which may explain the differences in its correlation with cachexia in some studies	[17]
Adipose tissue	Obesity Hyperlipidemia	Suppress appetite and regulate body weight Promote fat breakdown Anti-inflammatory effects Analogues as weight-loss therapeutic targets	Elevated levels associated with pathological obesity	In obese patients, weight loss induced by liraglutide or lorcaserin does not alter GDF-15 levels, suggesting its regulatory mechanism may be independent of known weight control pathways GDF-15 analogues have shown suboptimal weight-loss efficacy in clinical trials GDF-15 agonists and antagonists may be applied in the treatment of weight loss and cachexia, requiring precise regulation based on patients' metabolic status to avoid side effects such as cachexia-like symptoms in obesity therapy	[18, 19]
Muscle/Protein metabolism	Muscle atrophy Mitochondrial myopathy Idiopathic inflammatory myopathy	Acute stress protection after exercise Skeletal muscle repair after muscle injury Enhance muscle endurance Antibodies as intervention targets for sarcopenia	Accelerate muscle atrophy Lead to muscle strength decline and physical function deterioration	The effects of GDF-15 may be dose- and time-dependent: Acute high expression exerts protective effects, while chronic sustained expression leads to disease development	[20–22]
Bone composition	Osteoporosis Cancer with bone metastasis Rheumatoid arthritis	Long-term stimulation promotes bone remodeling: Inhibits osteoclast differentiation and promotes osteoblast formation	Overexpression accelerates bone loss: promotes osteoclast activation and inhibits osteoblast formation Associated with bone metastasis	Different expression levels exert completely opposite roles in disease development	[23]
Trace element - iron	Iron-deficiency anemia Thalassemia Aplastic anemia Pernicious anemia	Potential markers of iron metabolism disorders Monitor iron overload and its complications	Promote iron overload Cause iron-deficiency anemia Interfere with hepcidin	Currently, its correlation remains unclear, and systematic research is lacking	[24, 25]
MetS - IR	T2DM Central obesity PCOS	Increase insulin sensitivity Promote lipid breakdown Exert anti-inflammatory effects	May accelerate the progression of cardio-renal-MetS Promote metabolic disorders Associated with low-grade chronic inflammation	Some studies suggest that elevated GDF-15 is more a consequence of metabolic stress (e.g., energy deprivation, inflammation) than a protective mechanism, indicating its “beneficial effects” may reflect pathological states of metabolic disorders rather than improvement	[26–28]

**FIGURE 2:**
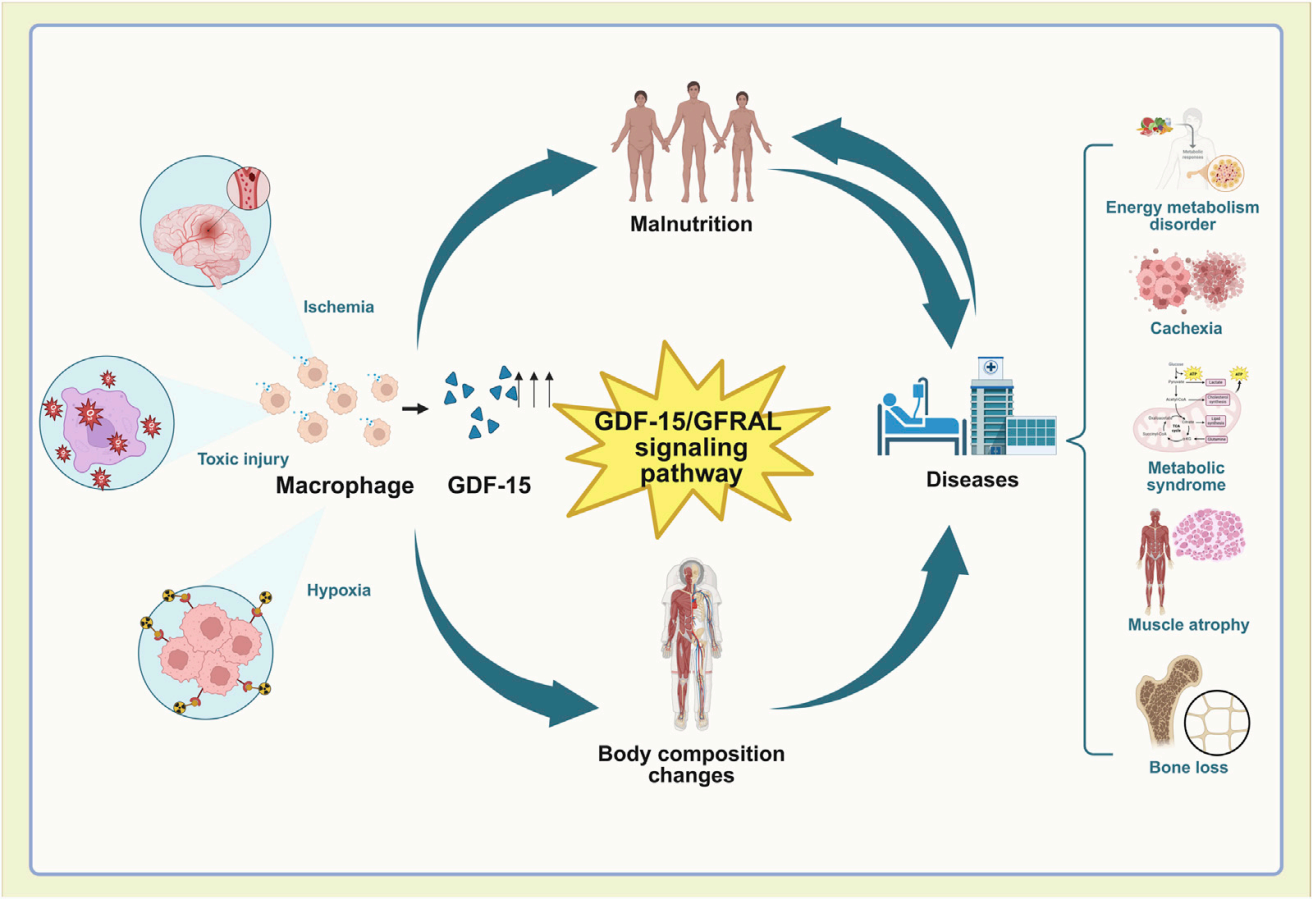
Schematic diagram of the GDF-15/GFRAL Signaling Pathway in pathophysiological cascades. This diagram illustrates the cyclic interplay of the GDF-15/GFRAL signaling pathway in driving malnutrition-related disorder progression. Macrophages activated by ischemia, toxic injury, hypoxia, and other stimuli secrete growth differentiation factor 15 (GDF-15), which triggers the GDF-15/GFRAL signaling pathway. This initiation sets off a cascade of events: induction of malnutrition, alteration of body composition, and promotion of disease development (e.g., energy metabolism disorders, cachexia, MetS, muscle atrophy, bone loss). These pathological changes in turn further exacerbate nutritional imbalance, forming a self-perpetuating vicious cycle between disease states and systemic malnutrition.

In recent decades, GDF-15 has received increasing attention in relation to multi-system diseases and the aging process. Furthermore, its expression corresponds to diverse indicators pertinent to underpinning nutritional status, serving as a good assessing surrogate or even a therapeutic target [13, 32] (Figure 2). However, the precise contribution of GDF-15 and its mechanism of action in multiple dimensions, such as energy metabolism, tissue repair, and inflammatory activity, has not been fully elucidated. There are still knowledge gaps with respect to the interplay between GDF-15 and other nutrition-related indicators along with its predictive value concerning early diagnosis, monitoring, and prognostication. Herein, we systematically synthesize relevant information derived from basic research, clinical studies, and translational evidence. This article sheds light on the molecular regulatory network of GDF-15 in the context of nutritional metabolism and disease development, clarifying its clinical implications as a novel diagnostic tool and assessing metric, and providing a theoretical basis and new strategy for the prompt intervention and personalized management. We present this article in accordance with the narrative review reporting checklist.

## Methods

We searched for literature with terms “nutrition and GDF-15,” “body composition and GDF-15,” “energy metabolism and GDF-15,” “appetite and GDF-15,” and others published in PubMed, Google Scholar and other search engines, encompassing articles in the English language up to 31st May 2025 (Table 2), with additional literature published in 2026 supplemented during the revision period.

**TABLE 2:** The search strategy summary.

Items	Specification
Date of search	1 May 2025 to 31 May 2025
Databases and other sources searched	PubMed, google scholar
Search terms used	Nutrition and GDF-15, body composition and GDF-15, energy metabolism and GDF-15, appetite and GDF-15, cachexia and GDF-15, muscle atrophy and GDF-15, MetS and GDF-15, older people and GDF-15, therapy and GDF-15
Timeframe	The search covered studies published up to May 2025
Inclusion and exclusion criteria	Only basic research, clinical information and translational evidence in English language were included. Case reports or studies lacking relevance were excluded
Selection process	Two independent reviewers conducted the research of studies independently and after the results were matched for the selection of those finally included in this review

## GDF-15 in energy metabolism and appetite

### GDF-15 in energy metabolism and appetite regulation

GDF-15 is significantly increased under acute complete starvation and positively correlated with triglyceride-rich lipoproteins, a trend more pronounced in patients with Relative Energy Deficiency in Sport, suggesting that it may be used as ‘a marker of energy metabolism’ [33]. In another study, intermittent fasting increases the level of GDF-15 in obese women by 5% compared with caloric restriction, but this change is not directly delivered to food intake, implying alternative pathways associated with energy metabolism regulation other than GFRAL [34]. Obesity represents a global health problem, whose pathogenesis can be attributable to the imbalance between energy metabolism and nutritional intake.

As an appetite-related stress signal, GDF-15 may possess multiple potentials in obesity: animal experiments have confirmed that recombinant GDF-15 can exert anti-obesity effects by inhibiting appetite and reducing body weight [35]. Although GDF-15 has been reported to lower body weight, circulating GDF-15 levels in obese patients are generally elevated [36]. This may reflect a compensatory action in which GDF-15 is accommodated to obesity-related metabolic disorders, such as insulin resistance (IR), and serves as a stress-response cytokine. However, long-term intensive metabolic derangement may dampen this compensation. It is shown that GDF-15 inhibits appetite and reduces food intake by activating the GFRAL signaling pathway in hindbrain neurons, thereby markedly reducing body weight and improving blood glucose in obese models [18, 37]. This pathway provides clearer causal evidence for GDF-15-mediated appetite suppression in animal models. Exogenous GDF-15 treatment can rapidly improve insulin tolerance in obese rats independent of body weight changes. In other words, GDF-15 may restore or even enhance insulin sensitivity in the liver and adipose tissue through mechanisms mediated by the β-adrenergic receptor regardless of GFRAL [26, 38]. Mechanistically, this protective effect involves the regulation of adipose tissue function and macrophage polarization [38]. In weight-loss interventions for rat models, the response of GDF-15 appears to be pleiotropic: bariatric surgery (e.g., sleeve gastrectomy) significantly increases GDF-15 levels in the circulation and gastrointestinal tissues, especially in those with metabolic syndrome (MetS)-like conditions; high expression of GDF-15 after surgery fosters energy balance, partially interpreting the phenomenon of weight loss postoperatively [39]. During exercise interventions, 60 min of moderate-intensity continuous intervention can increase the GDF-15 level among obese individuals alongside appetite suppression, and there is no energy compensation after exercise [40]. In other words, individuals do not compensate for the energy expended during exercise by increasing food intake afterward, thus helping to create a negative energy balance. Accordingly, preclinical studies have argued that GDF-15 analogues can become a potential therapeutic target against the global obesity epidemic [41]. Altogether, a line of evidence indicates that GDF-15 is dynamically regulated by energy status and correlates closely with starvation, obesity, exercise, and weight loss. Its causal role in suppressing appetite and reducing body weight is well established in animal studies via the GDF-15/GFRAL/rearranged during transfection (RET) axis, yet the mechanisms mediating these effects independently of this axis remain largely unknown. Therefore, elevated circulating GDF-15 should be interpreted as an integrated signal of energy deficit, lipid metabolism, inflammation, and tissue stress—not merely as an anorectic marker.

### GDF-15 in anorexia nervosa

As a key regulator of appetite and metabolic stress, GDF-15 has gained prominence in anorexia nervosa (AN)—a severe mental disorder characterized by significant weight loss, persistent dietary restriction, and disrupted energy balance [42]. Elevated GDF-15 levels are implicated in appetite suppression, weight loss, and early satiety—hallmarks of AN pathology.

Notably, serum GDF-15 levels are approximately 15% higher in acute AN patients than in healthy controls, and decrease significantly with refeeding and nutritional recovery [43]. This reversible pattern suggests that GDF-15 reflects nutritional state and metabolic stress rather than a fixed trait of AN; this finding simultaneously supports its sensitivity to hunger and malnutrition, positioning it as a potential marker of disease activity. Furthermore, high serum GDF-15 levels correlate with ghrelin, jointly predicting BMI reduction probably due to appetite suppression in AN patients [44]. This interaction may partially interpret persistent appetite suppression, but current evidence is insufficient to determine whether GDF-15 precedes or follows weight loss. A high GDF-15 level (>0.8 ng/mL) is also linked to fibroblast growth factor 21 (FGF21) metabolic changes and mitochondrial dysfunction, suggesting AN patients may be in a prolonged state of energy deficiency and metabolic stress [45]. The elevation of GDF-15 is associated not only with the pathology of AN but also with chronic metabolic stress. Exercise, high insulin levels, and certain metabolic drugs (e.g., metformin) can significantly induce GDF-15 expression in AN patients, mediated by insulin and glucagon’s coordinated regulation [46–48]. This highlights GDF-15’s sensitivity to energy balance and metabolic changes. Accumulating evidence suggests that GDF-15 acts on appetite-control centers in the brainstem, amplifying feelings of disgust and avoidance associated with hunger [49]. These studies reveal that elevated GDF-15 not only reflects nutritional status but may directly contribute to appetite inhibition through central appetite regulation. However, direct causality in AN remains uncertain, as appetite restriction is strongly shaped by psychiatric and behavioral factors. Collectively, the current evidence mainly supports GDF-15 as a dynamic marker of starvation, malnutrition and chronic metabolic stress in AN. Although GDF-15 may contribute to appetite suppression through brainstem and gut-brain pathways, its causal role remains difficult to separate from psychiatric drivers, excessive exercise, hormonal adaptation and low body weight.

### Mechanisms of GDF-15-induced anorexia

**FIGURE 3:**
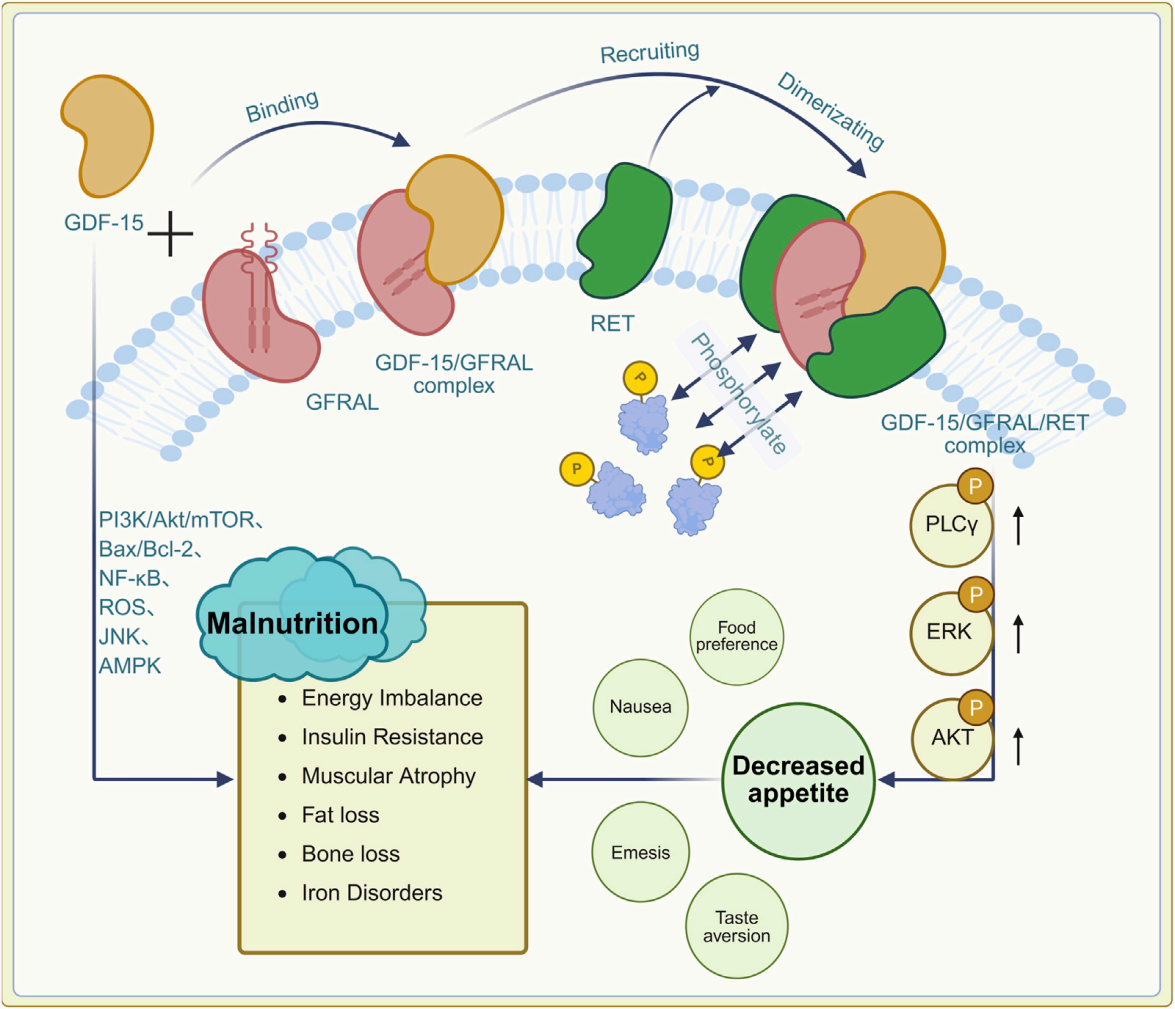
The molecular mechanism mediated by GDF-15 in malnutrition. The GDF-15/GFRAL/RET signaling pathway is one of the core pathways through which GDF-15 induces malnutrition. This diagram illustrates the downstream signaling network activated upon GDF-15 binding to the GFRAL receptor, which is primarily localized in neurons of the AP and NTS within the hindbrain, to form the GDF-15/GFRAL/RET complex, along with its pathophysiological effects: GDF-15 first binds to GFRAL to form a GDF-15/GFRAL complex, which then recruits and dimerizes with RET to form the ternary GDF-15/GFRAL/RET complex. This complex triggers the phosphorylation of PLCγ, ERK, and Akt, thereby inhibiting the activity of appetite-promoting neurons (e.g., AgRP/NPY neurons) in the medullary hindbrain while enhancing the excitability of anorectic neurons (e.g., POMC neurons), producing an anorectic effect. These signaling events lead to appetite suppression, manifested as nausea, vomiting, taste aversion, and altered food preferences, ultimately causing malnutrition indirectly by reducing energy intake. Additionally, GDF-15 activates multiple signaling pathways (PI3K/Akt/mTOR, Bax/Bcl-2, NF-κB, ROS, JNK, AMPK), culminating in malnutrition-related disorders such as energy imbalance, IR, muscle atrophy, adipose tissue loss, bone loss, and iron metabolism disorders.

Referring to the appetite regulation, GDF-15 has been regarded as ‘an anorectic factor’ and exerts an intrinsic function (Figure 3). The underlying mechanism of anorexia induced by GDF-15 has been broadly corroborated in the existing literature. GDF-15 activates its specific receptor GFRAL, which is primarily localized in neurons of the AP and nucleus of the solitary tract (NTS) within the hindbrain. This interaction induces anorectic action and subsequently triggers the phosphorylation of extracellular signal-regulated kinases 1/2 (ERK1/2), signal transducer and activator of transcription 3 (STAT3), AMP-activated protein kinase (AMPK) and PI3K/Protein kinase B (Akt) signaling pathways [6, 8, 18, 50]. This process inhibits hypothalamic orexigenic neuropeptide Y (NPY) neurons and stimulates the activity of anorexigenic proopiomelanocortin (POMC) neurons, and in turn leads to decreased appetite. The GDF-15/GFRAL/RET pathway is regarded as a canonical regulatory pathway for anorexia and resultant weight loss alongside a novel therapeutic target for cachexia [5, 51]. In addition, there are other mechanisms responsible for GDF-15-directed anorexia: GDF-15 activates the hypothalamic-pituitary-adrenal axis (HPA), leading to the secretion of corticotropin-releasing hormone (CRH) and glucocorticoids (GC), thereby leading to the appetite inhibition in patients with cachexia [52]. It is worth noting that GDF-15 can additionally influence the loss of fat and muscle. Tumor-bearing mice with overexpressed GDF-15 exhibited a significant anorexia phenotype, manifested with a continuous decrease in food intake [5]. This feeding inhibition indirectly causes metabolic chain reactions such as decreased fat mass and muscle atrophy (tibialis anterior and gastrocnemius), responsible for an overall weight loss of 28%.

### GDF-15 in disease-related cachexia and anorexia

Beyond its role in general appetite and energy homeostasis, GDF-15 has been implicated in a metabolic status characterized by profound weight loss and disordered energy metabolism, that is disease-related cachexia. This includes cancer cachexia and other types such as cardiac cachexia and chronic obstructive pulmonary disease (COPD)-associated cachexia.

Regarding pancreatic cancer and non-small cell lung cancer, elevated serum GDF-15 levels (>3.4 ng/mL, as reported by Suzuki H et al.) are associated with cachexia characterized by continuous weight loss, deterioration of physical condition, and excessive tumor burden [15, 53]. In patients with advanced cardiac failure, high GDF-15 levels (>1.2 ng/mL) may pinpoint a higher incidence of cachexia and predict a higher incidence of adverse events [54]. In this regard, it is plausible to improve cardiac function and delay the progression of cachexia by blocking the GDF-15 pathway in mice with heart failure-associated cachexia [55]. In childhood cancers, GDF-15 upregulation not only predicts the risk of chemotherapy-related nausea, vomiting, anorexia, and weight loss, but also is connected to anthracycline cardiotoxicity and increased mortality [56]. Several clinical studies have verified that GDF-15 in anorexic cancer patients (median: 1.2 ng/mL) is significantly higher than that in non-anorexic cancer patients (median: 0.8 ng/mL) and healthy controls (median: 0.6 ng/mL) [57]. Notably, GDF-15 in patients with cachexia is positively correlated with the magnitude of anorexia, conferring its potential as a biomarker contextually [5, 53, 54, 57]. Besides, cancer cachexia triggers intractable anorexia and weight loss by activating the brainstem GDF-15/GFRAL nausea or vomiting center, and treatment-related nausea or vomiting due to chemotherapy (e.g., platinum drugs) inducible GDF-15 secretion, where all antiemetic drugs cannot effectively prevent and treat this discomfort [58]. Collectively, GDF-15 is involved in cancer cachexia development. Current observational and cross-sectional evidence shows that elevated GDF-15 levels correlate positively with cachexia severity, anorexia, chemotherapy-induced nausea, and poor prognosis, yet mechanistic validation studies are still lacking. The current view favors that GDF-15 acts synergistically with tumor burden, systemic inflammation, and treatment toxicity rather than as an independent cause. GDF-15 should be defined as a sensitive marker and amplifier of disease-related anorexia, not a single etiological agent of cancer cachexia.

Intriguingly, the anorexic effect pertinent to GDF-15 has a dual role regarding obesity and cachexia. In obesity, high levels of GDF-15 may potentiate appetite control and weight reduction; in the circumstance of cachexia, GDF-15 may aggravate this detrimental status, accelerate disease progression, and reduce the quality of life and survival time. Its effect on metabolism may be diminished under long-term intensive metabolic stress due to obesity [19]. On the other hand, sustained high levels of GDF-15 lead to uncontrollable energy consumption [15, 59]. Notably, most evidence is derived from rodent models, with marked differences in GFRAL expression and downstream signaling between humans and mice. The dual role of GDF-15 in obesity and cachexia suggests its function is highly context-dependent, yet a clear dose-response relationship has not been established in humans. Therefore, GDF-15 should be recognized as a context-dependent molecule: it may act as an anorexigenic mediator under specific experimental conditions, but in most clinical populations, it more frequently serves as a biomarker of metabolic stress and disease burden.

## GDF-15 and muscle

### Physiological role of GDF-15 in exercise and muscle repair

GDF-15 appears to have multifaceted roles in muscle status that differ between physiological adaptation and pathological stress. Under physiological conditions, GDF-15 regulates exercise stress and skeletal muscle health. Both acute high-intensity exercise and chronic moderate-intensity exercise can upregulate the expression of GDF-15 in humans and mice [12, 60–62]. Its circulation level peaks at 120 min after stopping exercise and decreases toward baseline 48 h after exercise in trained individuals (before exercise: 0.6 ± 0.1 ng/mL; end of exercise: 2.3 ± 0.5 ng/mL; 48 h post-exercise: 0.9 ± 0.2 ng/mL, as reported by Tchou I et al.) [12, 61]. Although animal experiments demonstrate that acute exercise is insufficient to alter circulating GDF-15, a marked increase of GDF-15 in muscle tissue may be protective against exercise stress response [60]. In another study, prolonged endurance exercise increases circulating GDF-15 levels comparable to those under pathological stress in healthy adults; further animal experiments confirmed that this process was accompanied by increased GDF-15 expression in the liver, skeletal muscle, and myocardia [63]. Mechanistically, GDF-15 may inhibit the differentiation of fast muscle fibers and promote the formation of slow muscle oxidative fibers by activating the Akt/mTOR pathway to strengthen muscle endurance [64]. Of note, GDF-15 induction during exercise is sensitive and dependent on exercise intensity and time; that is, short-term (≤1 month) moderate-intensity training is capable of increasing GDF-15 expression, in contrast to short-term high-intensity interval training and long-term (>6 months) regular exercise training, which appear to have only limited effects on GDF-15 [65]. This pattern suggests that GDF-15 may reflect whether exercise load exceeds the current adaptive capacity, rather than simply indicating training benefit itself. Intriguingly, the upregulation of GDF-15 after exercise is more pronounced in individuals with a sedentary lifestyle [62]. In terms of striated muscle repair, GDF-15 strengthens muscle regeneration via multiple mechanisms. Taking myocardial injury as an example, overexpressed GDF-15 after myocardial injury can reduce cell damage by up-regulating the expression of telomerase reverse transcriptase and activating the AMPK signaling pathway [66]. Meanwhile, GDF-15 effectively tackles inflammatory response in the process of satellite cell activation, myoblast proliferation and muscle regeneration, and accelerates muscle fiber remodeling and functional recovery in mice [67].

The physiological effect of GDF-15 represents a product of exercise-induced muscle protective response along with a key mediator involved in inhibiting fast muscle differentiation, enhancing mitochondrial function, promoting cell regeneration, and participating in muscle adaptive reconstruction. However, current evidence in humans is mostly observational with limited mechanistic research, leaving its causal role in humans unclear. Given the complex origin and context-dependent function of GDF-15, its exercise-induced elevation is more likely a concomitant phenomenon of adaptive muscle remodeling rather than an active driver.

### Pathological role of GDF-15 in muscle dysfunction

From a pathological view, GDF-15 is related to the symptoms, severity, and prognosis. GDF-15 may serve as a novel biomarker for sarcopenia and frailty. Accumulating cross-sectional evidence has unraveled that higher GDF-15 (>1.2 ng/mL, as reported by Herpich C et al.) is associated with muscle dysfunction, sarcopenia, frail phenotype, decreased physical function, and poor prognosis [20, 68–71]. A sarcopenia index on the basis of GDF-15 and myostatin also exhibits excellent predictive performance in patients undergoing cardiovascular surgery [69]. Furthermore, GDF-15 is negatively correlated with skeletal muscle index, handgrip strength (HGS), knee extension strength and walking speed, but positively correlated with myostatin. GDF-15 has also been proven to be associated with the deterioration of muscle status. In patients with COPD, serum GDF-15 levels are negatively correlated with various metrics concerning muscle quantity, strength, and function [71]. Accordingly, patients with COPD and concurrent sarcopenia have significantly higher GDF-15 levels than controls. In patients with osteoporosis, the abnormal expression of GDF-15 is closely related to muscle homeostasis and bone metabolism imbalance. Muscular expression of GDF-15 mRNA is in alignment with inducible inflammatory factors (i.e., TNF-α and IL-1β), but negatively correlated with areal bone mineral density and distal radius bone mass, highlighting muscle-derived GDF-15-dictated inflammation to instigate obvious bone loss [72]. In addition, GDF-15 derived from muscle may be more critical than circulating GDF-15 to modulate muscle mass and function, and a paracrine pattern may account for this tissue specificity [72]. This may be because the former reflects the local musculoskeletal microenvironment, whereas the latter more often represents systemic stress. These findings jointly reveal that GDF-15 is a pivotal molecule connecting muscle-bone metabolism, and its abnormality may aggravate the occurrence of muscle atrophy and osteoporosis through synergistic inflammatory response and regional tissue action.

In mitochondrial myopathies, elevated GDF-15 levels are suggestive of muscle damage and mitochondrial dysfunction [73]. GDF-15 neutralizing antibody treatment significantly improves the metabolic phenotype of polymerase γ (POLG) mutant mitochondrial myopathy mice, as evidenced by increased body weight, muscle mass, skeletal muscle maximum strength alongside exercise endurance [21]. Mechanistically, the beneficial effects of GDF-15 neutralization are linked to the reversal of transcriptional dysregulation of genes ascribed to autophagy and proteasome signaling. This treatment also appears to dampen GC signaling by suppressing circulating corticosterone levels in the POLG animals [21]. GDF-15 is also associated with disease severity and prognosis of muscle metabolism-related diseases. In idiopathic inflammatory myopathy and juvenile dermatomyositis, GDF-15 level is significantly increased, and positively correlated with multiple disease activity indices (e.g., Disease Activity Score, skin/muscle score and Children Myositis Assessment Scale) [22]. There exists a distinct pattern between the active and remission stages of the disease, since a discriminative capability of GDF-15 has been addressed. In addition, GDF-15 is positively correlated with muscle injury markers (creatine kinase), immune activation indicators (neopterin), and vascular pathological markers (nailfold capillary abnormalities), implicated in the disease process [22]. These findings support GDF-15 as a comprehensive biochemical indicator for assessing disease activity and monitoring progression. Under pathological conditions, elevated GDF-15 is consistently associated with sarcopenia, muscle dysfunction, inflammatory myopathies, and mitochondrial muscle disease. However, most human evidence remains observational, making it unclear whether GDF-15 is a pathogenic driver or merely a marker of tissue damage. In summary, while GDF-15 is reasonably positioned as a “cross-disease marker of muscle assessment,” it should not be regarded as a definitive pathogenic factor in human muscle disorders.

### Mechanisms of GDF-15 in muscle atrophy

**FIGURE 4:**
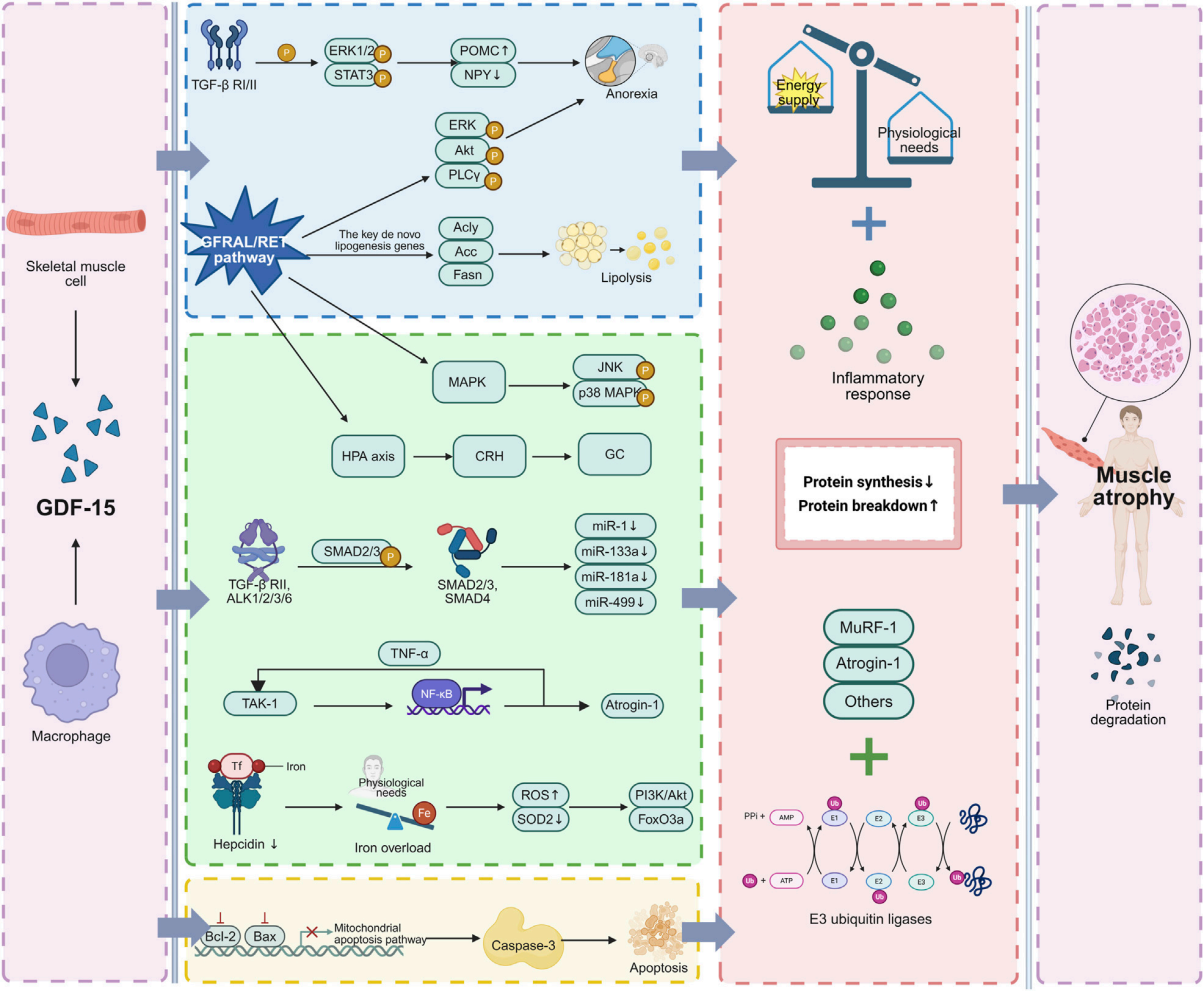
Multiplex pathway mechanisms of GDF-15 in mediating muscle atrophy. The figure depicts the molecular and physiological cascade of GDF-15-induced muscle atrophy. Secreted by skeletal muscle cells and macrophages, GDF-15 participates in the regulation of muscle metabolism through multiple signaling pathways (1) Energy Imbalance Mechanism: By activating the GFRAL/RET pathway, GDF-15 triggers ERK/Akt/PLCγ signaling and suppresses appetite via POMC/NPY-related neuroendocrine regulation. Simultaneously, it suppresses the expression of lipid synthesis-related genes (e.g., Acly, Acc, Fasn) to promote white adipose tissue breakdown, disrupting energy homeostasis and indirectly contributing to muscle atrophy. (2) Ubiquitin-Proteasome System Mechanism: GDF-15 activates the HPA axis/CRH/GC, MAPK/JNK, SMAD2/3/miRNA, MAPK/JNK/p38, and TNF-α/NF-κB pathways, synergistically up-regulating atrophy-related genes (e.g., atrogin-1) and E3 ubiquitin ligases. It further stimulates the proteasome system via the ROS/PI3K/Akt/FoxO3a pathway, accelerating protein degradation and shifting muscle metabolism toward catabolism. (3) Mitochondrial Apoptosis Mechanism: GDF-15 induces the Bcl-2/Bax/Caspase 3 cascade, triggering mitochondrial-mediated apoptosis and reducing muscle cell populations. These interconnected pathways collectively alter energy balance, amplify inflammatory responses, and activate proteolytic systems, ultimately leading to muscle atrophy and protein degradation.

Emerging evidence has delineated multiple pathways underlying GDF-15-mediated muscle atrophy, involving central appetite regulation, endocrine stress, lipid metabolism, and inflammation [5, 17, 51]. The proposed interpretations are depicted as follows: (1) Central activation of the brainstem GDF-15/GFRAL/RET pathway and downstream phosphorylated ERK/Akt/Phospholipase C (PLC) signals, mainly in the AP and NTS, suppresses appetite and indirectly promotes muscle wasting in cachectic mice [5, 51]; (2) GDF-15 influences CRH neurons via brainstem GFRAL neurons and their ascending projections, thereby activating the HPA axis and promoting the release of GC. Elevated GC further upregulates the expression of E3 ubiquitin ligase such as muscle-specific RING finger protein 1 (MuRF-1) and muscle atrophy F-box protein 1 (atrogin-1), drives the decomposition of muscle proteins, reduces the diameter of myotubes, and causes muscle atrophy in mice [74–76]; (3) At the peripheral muscle level, GDF-15 activates atrogin-1/MuRF-1 expression through the TGF-β-activated kinase 1 (TAK-1)/nuclear factor kappa B (NF-κB) pathway, and curtails a range of miRNAs (i.e., miR-1, miR-133a and miR-499) in muscle, resulting in decreased muscle mass and muscle atrophy in mice [74, 75]; (4) The activation of GFRAL-RET pathway suppresses the expression of lipid synthesis-related genes [i.e., ATP citrate lyase (Acly), acetyl-CoA carboxylase (Acc), and fatty acid synthase (Fasn)] in the adipose tissue. GDF-15 triggers lipolysis in the adipose tissue independently of anorexia, leading to a decrease in fat and muscle mass/function in tumor-bearing mice [51]; (5) High GDF-15 inhibits the expression of hepcidin and mediates iron overload by regulating forkhead box protein O3a (FoxO3a) expression through the reactive oxygen species (ROS)/PI3K/Akt axis in humans; iron overload itself has also been reported to independently induce muscle atrophy [77, 78]. Additionally, inflammation may also leverage a complicated impact on GDF-15 and muscle metabolic disorders. Elevated GDF-15 levels are significantly correlated with muscle injury markers (creatine kinase) and proinflammatory cytokines (TNF-α, IL-1β), implying that it may be involved in the muscle degeneration challenged by inflammatory insult [22, 72, 79]. It is worth noting that muscle-derived rather than circulating GDF-15 may be more relevant to local muscle mass and function, and its mechanism may involve the activation of core signaling pathways like NF-κB; meanwhile, chronic low-grade inflammation induces sustained high expression of GDF-15, which in turn promotes muscle atrophy [72, 79]. Until now, the relationship between GDF-15 and inflammation in the context of muscle metabolic disorders is still under intensive investigation [73]. Figure 4 demonstrates a mechanistic framework that distinguishes central indirect mechanisms, like anorexia and HPA-axis activation, from peripheral or systemic mechanisms, such as muscle proteolysis, adipose lipolysis, inflammation, and iron dysregulation.

These findings suggest that the role of GDF-15 in muscle homeostasis appears to be multifactorial, involving protein synthesis/degradation rebalance, innervation regulation, and a potential link with inflammation, metabolism, and redox balance. It should be noted that GFRAL is expressed almost exclusively in the brainstem. Therefore, mechanisms involving the GDF-15/GFRAL/RET axis (e.g., appetite suppression and HPA axis activation) are centrally mediated. Whether GDF-15 acts directly on peripheral skeletal muscle via GFRAL-independent pathways remains an open question, as current evidence is largely derived from correlative human studies or indirect animal models. The role of GDF-15 is neither simply protective nor detrimental; rather, it depends on biological context, concentration, duration, and tissue origin. Transient and moderate elevation may promote muscle adaptation and regeneration, whereas sustained high levels indicate or contribute to wasting conditions. It is plausible to use GDF-15 as a trans-disease marker of muscle status, but its direct pathogenic role and therapeutic potential should be judged on a disease- and intervention-specific basis.

## GDF-15 and metabolic syndrome

There is a complex interplay between GDF-15 and MetS. GDF-15 is linked to various pathological entities such as obesity, IR, atherosclerosis, dyslipidemia, inflammation and hypertension, contributing to the metabolic homeostasis maintenance. A number of cross-sectional studies have depicted that serum GDF-15 levels in MetS patients are significantly elevated and positively correlated with MetS components (e.g., hyperglycemia, central obesity) [27]. Studies have shown that the ratio of GDF-15 to adiponectin may be a surrogate for MetS with incremental diagnostic efficacy [80].

### GDF-15 in obesity and adipose metabolism

#### Clinical and preclinical evidence linking GDF-15 to obesity

Circulating GDF-15 levels have been reported to be significantly elevated in obesity, as demonstrated in obese mouse models [35]. In overweight and obese people, waist circumference and waist-to-height ratio positively correlate with GDF-15 level, while an opposite trend is observed in lean people, implying a role for GDF-15 in regulating central obesity [81]. GDF-15 embraces a complex multi-tissue synergy dynamically modulating adipose tissue, whose expression is sensitive to nutritional status. In normal weight animal models, a short-term high-fat diet can significantly suppress GDF-15 mRNA expression in white and brown adipose tissue [36], whereas a ketogenic diet can reduce energy intake and body weight, accompanied by increased GDF-15 levels [82]. These diet-responsive changes reinforce the adaptability of GDF-15 in lipid metabolism and underscore its importance in compensatory mechanisms during dietary interventions.

#### Dynamic tissue sources of GDF-15

Interestingly, multiple studies in mice have demonstrated that the source of circulating GDF-15 changes dynamically according to metabolic status. In the early phases of obesity and type 2 diabetes mellitus (T2DM), adipose tissue-resident macrophages serve as the primary secretory source of GDF-15 [83]. Moreover, genetic ablation of GDF-15 in these macrophages reduces plasma GDF-15 levels and worsens obesity-related conditions [83]. As metabolic dysfunction progresses to metabolic dysfunction-associated steatohepatitis (MASH), although local biopsy of adipose tissue shows no significant fluctuations in GDF-15 mRNA levels, the liver assumes a dominant role in GDF-15 secretion [35, 82, 83]. Specifically, the liver mediates the transcription and production of GDF-15 by activating the peroxisome proliferator-activated receptor γ (PPARγ) and binding to its regulatory sites [82]. Moreover, during acute metabolic stress or dietary transitions, gastric GDF-15 is a critical contributor to circulating GDF-15 levels [39, 84]. Notably, a seeming contradiction emerges: although adipose tissue macrophages are a key source in early obesity, whole adipose tissue itself contributes minimally to circulating GDF-15, as demonstrated in GDF-15 knockout mice [35, 83]. This discrepancy highlights the need to distinguish between specific macrophage populations and total adipose tissue when interpreting GDF-15 dynamics. These findings underscore that GDF-15 tissue origins dynamically shift across metabolic states, with organ-specific contributions forming a tightly orchestrated regulatory network to shape GDF-15 secretion.

#### Mechanistic actions of GDF-15 in obesity-related metabolism

**FIGURE 5:**
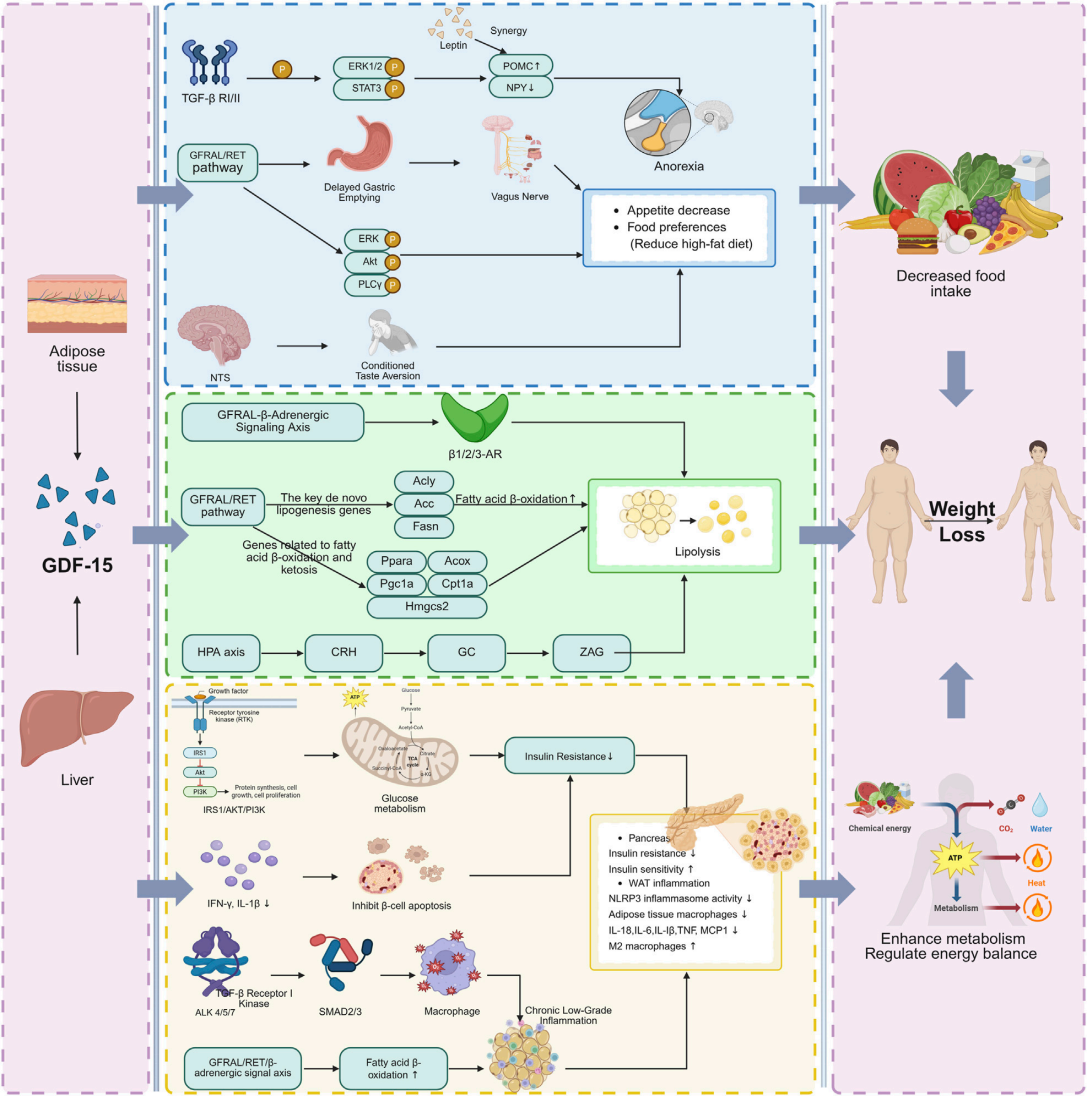
Multiplex pathway mechanisms of GDF-15 in mediating weight loss. The figure illustrates the multifaceted mechanisms through which GDF-15 promotes weight loss by regulating metabolism and appetite. Under stress conditions, increased secretion of GDF-15 from tissues such as skeletal muscle and liver exerts its effects via three core pathways (1) Neuro-Gastrointestinal Axis: GDF-15 activates the GFRAL/RET signaling pathway, upregulating POMC while suppressing NPY, stimulating the vagus nerve via the NTS, thereby inhibiting appetite, delaying gastric emptying, inducing conditioned taste aversion, reducing high-fat diet intake, and lowering overall food consumption, ultimately leading to a negative energy balance and indirect weight loss; (2) Adipose Tissue Remodeling Axis: GDF-15 promotes fat breakdown and directs weight loss by activating the GFRAL/β-adrenergic, GFRAL/RET, and HPA signaling axes, as well as enhancing the expression of lipolysis-related genes; (3) Metabolic Regulation Axis: GDF-15 improves glucose metabolism and reduces IR by activating the IRS1/Akt/PI3K pathway, suppressing NLRP3 inflammasome activity via mediating SMAD2/SMAD3 signaling through TGF-β RI receptor kinases (ALK 4, ALK 5, and ALK 7), and blocking pro-inflammatory cytokines (IFN-γ and IL-1β)-induced pancreatic β-cell apoptosis, thereby enhancing systemic insulin sensitivity and reducing adipose tissue infiltration. These pathways work synergistically to reduce food intake, decrease body weight, and optimize energy homeostasis.

As a pleiotropic cytokine, GDF-15 plays a complex role in the pathophysiological process of obesity and related metabolic diseases. The mechanism involves the following aspects: (1) Appetite management: Studies have shown that exogenous administration of GDF-15 can significantly reduce food intake and body weight in obese animal models, also verified in rodents and primates [6, 50, 85]. The anorectic effect of GDF-15 mainly depends on the GFRAL/RET pathway in the brainstem AP/NTS region, activating downstream signaling such as ERK, Akt and PLCγ to reduce appetite and energy intake [6, 7, 41, 86, 87]. Meanwhile, GDF-15 indirectly modulates the hypothalamic appetite regulatory network by upregulating POMC and inhibiting NPY expression, which may synergize with leptin signaling to further strengthen central anorectic effects in obese mice [19]. Under certain animal experimental conditions, GDF-15 acts on the NTS to induce vomiting and promote conditioned taste aversion [88, 89], reducing preference for high-fat diets [90]. At the peripheral level, GDF-15 may delay gastric emptying and enhance satiety signals via the vagus nerve to reduce food intake [85, 88, 91]. (2) Energy metabolism and IR: Mouse studies have shown that GDF-15 improves obesity-related IR by enhancing Insulin receptor substrate 1 (IRS1)/PI3K/Akt insulin signaling and promoting glucose metabolism in adipose tissue [92]. Meanwhile, GDF-15 may reduce inflammatory cytokines such as Interferon-γ (IFN-γ) and IL-1β, inhibit pancreatic β-cell apoptosis, preserve β-cell function, and improve insulin sensitivity [93, 94], thereby contributing to the improvement of obesity-related IR and body weight regulation [35, 38]. (3) Inflammation regulation: In both mouse models and human studies, GDF-15 exhibits anti-inflammatory properties, alleviating chronic low-grade inflammation associated with obesity [83]. In adipose tissue, GDF-15 transmits activating signals to mothers against decapentaplegic homolog 2 (SMAD2) and SMAD3 via the TGF-β receptor I, including activin receptor-like kinase (ALK) 4, ALK 5, and ALK 7, upregulates oxidative function in macrophages, leading to M2-like polarization and reversal of IR in mice [95]. In addition, GDF-15 may enhance fatty acid β-oxidation via the GFRAL/RET/β-adrenergic signaling pathway, improving adipose metabolic homeostasis and indirectly reducing chronic low-grade inflammation [96]. These anti-inflammatory effects help mitigate obesity-related systemic inflammation and, indirectly, improve adipocyte function. (4) Adipose tissue remodeling: GDF-15 may increase fatty acid oxidation through the GFRAL–β-adrenergic-dependent signaling axis, thereby enhancing adipose tissue catabolism, promoting fatty acid utilization, and reducing fat accumulation [97]. At the metabolic gene level, GDF-15 downregulates *de novo* lipogenesis-related genes, including Acly, Acc, and Fasn, thereby suppressing fatty acid synthesis; meanwhile, it upregulates metabolic genes such as Ppara, Pgc1a, and Cpt1a, promoting fatty acid β-oxidation, ketogenesis, and energy expenditure [98, 99]. In addition, GDF-15 may promote lipolysis and energy mobilization through the HPA axis and CRH/GC/ZAG-related signaling [52]. GDF-15 undergoes lysosomal degradation via the autophagy pathway, inhibits adipocyte differentiation and reduces fat accumulation through homologous-pairing protein 2 (HOP2) -mediated inhibition of CCAAT/enhancer binding protein α (C/EBPα) expression [36, 83]. In a mouse model of experimental diabetic cardiomyopathy, GDF-15 reduces cardiovascular risks associated with obesity by attenuating endothelial dysfunction, atherosclerotic lesions, and myocardial fibrosis [38, 100] (Figure 5). In summary, GDF-15 has become a potential target against obesity and its complications by harnessing multiple pathways, that is, inflammation inhibition, oxidative stress relief and metabolic remodeling [37, 41].

Notably, nearly all causal evidence is derived from animal models, and its extrapolability and explanatory power in humans remain to be validated. Although the tissue origin of GDF-15 shifts dynamically with metabolic status, the functional significance of these changes in humans has yet to be fully elucidated. Consequently, the causal role of GDF-15 in human obesity and metabolic regulation remains unclear. Collectively, current data support GDF-15 as a dynamic stress factor closely associated with obesity and metabolic disorders, rather than a mere marker of adiposity.

### GDF-15 with IR and diabetes

#### Clinical associations among GDF-15, IR, and diabetes

The relationship between GDF-15 and IR has been extensively discussed in the context of diabetes [101–103], obesity [26] and polycystic ovary syndrome (PCOS) [104, 105]. Observational studies suggest that elevated serum GDF-15 can differentiate T2DM (1.5 ± 0.8 ng/mL) and non-diabetic patients (0.8 ± 0.4 ng/mL) [101–103]. In patients with T2DM, elevated GDF-15 levels are in close relation to fasting blood glucose, glycated hemoglobin, fasting insulin, and Homeostatic Model Assessment for IR (HOMA-IR) [103]. The human genetic polymorphism studies confirm that the correlation between GDF-15 and diabetes is also verified: the GC + CC genotype of the GDF-15 gene rs1054564 polymorphism is associated with an increased risk of T2DM [102]. Furthermore, prospective cohort and cross-sectional studies have shown that high levels of GDF-15 are also predictive of a significant increase in diabetes risk complicated with tumors, cardiovascular disease [106, 107], along with a range of microvascular pathologies, incorporating diabetic nephropathy [108, 109], diabetic retinopathy and neuropathy [110, 111].

#### Sex and ethnic differences

In human studies, the impact of GDF-15 on diabetes demonstrates notable gender differences but appears largely consistent across ethnic backgrounds. Specifically, obese female patients with diabetes (0.7 ± 0.3 ng/mL) exhibit lower circulating levels of GDF-15 compared to their male counterparts (1.5 ± 0.8 ng/mL) [112], suggesting a sex-dependent modulation of GDF-15 expression in the context of obesity and metabolic dysfunction. In contrast, studies comparing patients with T2DM from South Asia and Europe found no significant difference in GDF-15 levels, indicating its role goes beyond racial or geographical factors [113]. These findings suggest that sex may influence circulating GDF-15 levels, possibly through differences in sex hormones, fat distribution, body composition, or inflammatory status. However, the current evidence remains limited and largely descriptive. Most studies were not specifically designed to test sex-specific mechanisms, and therefore no firm conclusion can yet be drawn regarding sex-dependent biological functions of GDF-15 in diabetes. Future studies should include sex-stratified analyses and mechanistic validation.

#### Potential mechanisms linking GDF-15 to IR

The mechanism of GDF-15 to improve IR can be found as follows: (1) Receptor-dependent pathway: In obese rodents, without causing weight loss, activation of the GDF-15-GFRAL axis increases β-adrenergic signaling to enhance insulin activity in the liver and adipose tissue, in addition to inhibiting endogenous glucose production and promoting glucose uptake in white and brown adipose tissue [26]. (2) AMPK activation: GDF-15 improves IR and hepatic steatosis by restricting appetite and reducing inflammation, increasing thermogenesis and lipid catabolism alongside maintaining AMPK activity [28]. (3) Inflammation modulation: GDF-15 levels positively align with pro-inflammatory factors (e.g., IL-6, C-reactive protein) in diabetes [114], and possess an indirect effect on IR by counteracting chronic inflammation (e.g., macrophage polarization) to protect β cells from apoptosis and resulting islet dysfunction [38, 115]. In animal models of diabetic nephropathy, overexpression of GDF-15 can significantly reduce several cytokines, and prevent the ubiquitin degradation of IKK by inhibiting the expression of neural precursor cell expressed developmentally downregulated 4-like (NEDD4L) and resulting NF-κB pathway [79]. (4) Gene regulation: GDF-15 is regulated by miR-181b-5p, miR-330-3p and SMAD Family Member 7 (SMAD7), involved in the process of IR in visceral adipose tissue and peripheral blood mononuclear cells in patients with diabetes [101]. (5) Oxidative stress: In MetS patients, GDF-15 is highly correlated with ROS levels, and the upregulation of GDF-15 mRNA is accompanied by a decrease of antioxidant nuclear Nrf-2, suggesting that GDF-15 participates in metabolic regulation by controlling oxidative equilibrium [27]. Regarding potential therapeutic targets, studies have found that antidiabetic drugs such as metformin can increase circulating GDF-15 levels to improve IR as a novel medicinal attribute [28]. GDF-15 analogues or receptor agonists are under investigation, which may ameliorate IR by regulating metabolism and inflammation to counteract MetS [28, 115]. Notably, only an evident association between GDF-15 and IR or glucose dysregulation has been established; this evidence suggests that elevated GDF-15 represents ‘a bystander’ of metabolic stress rather than a direct pathogenic factor, and the causality is supposed to be verified by well-designed genetic and longitudinal studies.

## GDF-15 and malnutrition in older people

### GDF-15 in aging and cellular senescence

As a pleiotropic molecule, GDF-15 plays a crucial role in the development and progression of aging and age-related diseases. GDF-15 is markedly expressed in senescent cells, connected to actual age, the formation of epigenetic aging markers (e.g., DNAm GrimAge, DNAm PhenoAge, Hannum, and Zhang clocks), the decrease in telomerase activity, and the production of a senescence-associated secretory phenotype [116, 117]. Its mechanism of action involves multiple biological processes based on distinct biological backgrounds and actual ages [118]. Studies have unraveled that GDF-15 participates in heterogeneous biological pathways including but not limited to energy homeostasis, stress response and inflammatory regulation [116, 119, 120]. Previously, GDF-15 was identified as one predominant marker pertinent to aging and frailty-related genes and proteins, pinpointing its significance in accelerating the aging process. More recently, many studies have addressed that GDF-15 abnormality can reflect the nutritional status and physical function among older people. Circulating GDF-15 levels are not only positively correlated with actual age [23], but also with biological age-related markers [117], malnutrition [32, 121] and physical decline indicators [69, 119, 122]. Aforesaid evidence has jointly regarded GDF-15 as an important molecular node of cell senescence, body senescence and clinical phenotypes. Its multifactorial roles cover the regulation of aging at the cellular level and maintain organ function, therefore providing potential monitoring targets and therapeutic windows against aging-related malnutrition.

### GDF-15 in physical decline and frailty in older people

As a core biomarker of aging, the serum level of GDF-15 parallels increased age, especially in subjects over 60 years [23]. Older people are often associated with varying magnitudes of anorexia, characterized by decreased appetite and reduced food intake, as well as an increased risk of malnutrition [123]. In the acutely hospitalized older population, elevated GDF-15 levels are linked to deterioration of nutritional status assessed by Mini Nutritional Assessment-Short form (MNA-SF), loss of appetite assessed by the simplified nutritional appetite questionnaire, and decreased physical function in terms of various assessment (i.e., HGS, 30-s chair stand test, and gait speed), independent of age, gender, C-reactive protein levels, metformin or immunosuppressive therapy [121]. The baseline GDF-15 predicts the patients' later loss of appetite and progressive weight loss [124]. In patients undergoing hemodialysis, GDF-15 embraces moderately predictive utility for malnutrition screening [125]. Additionally, GDF-15 may be a potential regulator of malnutrition in older people, and its mechanisms of action and clinical relevance are age- and gender-dependent. On one hand, the fasting GDF-15 level and its dynamic changes are negatively correlated with appetite in older people rather than young women [126], partially interpreting the phenotype of ‘aging anorexia’, known as age-related loss of appetite responsible for insufficient intake of protein and micronutrients alongside increased malnutrition risk [123]. Elevated serum GDF-15 concentration implicates the deterioration of nutritional status in older women, manifested as a decrease in MNA-SF score, and this association persists even after excluding patients with cardiovascular disease or diabetes [32]. On the other hand, there is also a gender disparity pertaining to the connection between GDF-15 and physical function parameters such as HGS and knee extension [68]. This gender disparity may be due to differences in hormone levels or in metabolic stress responses [127]. GDF-15 as a predictor of physical functional decline in older people is being continuously validated. A cross-sectional study enrolling healthy people ≥60 years implicates that high GDF-15 levels are significantly associated with physical defects such as lung dysfunction and HGS decrement, especially in men [117]. Longitudinal studies show that GDF-15 can predict the onset of frailty in older patients hospitalized due to cardiovascular disease, with an increased likelihood of around 4 times [122]. Meanwhile, it is independently associated with numerous body dysfunction indicators, including low HGS, low gait speed, long timed-up-and-go time and scores of lower extremity function [69], further validated in another pre-frail older population [128]. Dynamic observation demonstrates that GDF-15 may be one of the molecules mediating the link between physical activity and later body weight changes in older people [119]. Taken together, GDF-15 may serve as an important surrogate to early identify frailty risk in older people. These findings collectively indicate that GDF-15 is not only a sensitive biomarker of age-related functional decline, but also a key molecule in the pathogenesis of malnutrition and physical decline.

As a multifaceted biomarker, GDF-15 has shown clinical significance in the evaluation of aging, physical function, and nutritional status. Its measurement is more readily available to implement than the epigenetic clock for promptly estimating age-related functional decline and malnutrition [117]. Nevertheless, the predictive efficacy of GDF-15 in older populations still requires further in-depth clinical research for conclusive verification. Other research topics should focus on elucidating the molecular mechanism of gender differences, in particular, the biological basis of women’s sensitivity to GDF-15 administration [32, 117].

## Clinical application and therapeutic potential

Accumulating evidence indicates that GDF-15 has predictive utility across a variety of clinical scenarios. GDF-15 is an effective driver of anorexia and weight loss, proposed in several studies to provide new therapeutic opportunities for obesity and cachexia. This molecule inhibits appetite in the context of obesity but aggravates energy consumption in cachexia, whose bodily impact is completely opposite, requiring delicate regulation of its activity [37]. In terms of obesity treatment, GDF-15 analogues or GFRAL receptor agonists can be prescribed as novel weight-loss drugs. At present, those are still in the early development stage, aiming to help resist obesity by regulating appetite and metabolism. However, there is still a long distance pertinent to integral clinical application [7, 41]. Technical problems should be acknowledged as the short half-life and aggregation tendency of GDF-15 [129]. The half-life of natural GDF-15 in mice and non-human primates is approximately 3 h, and it is susceptible to proteolysis, dramatically restraining the treatment delivery [85]. The addition of recombinant modifications, such as the fragment crystallizable regions of human serum albumin and Immunoglobulin G Fc region (IgG Fc), significantly prolongs the half-life of GDF-15 without hindering physiological anorexia and weight loss [85, 129]. Animal studies denote that a combination of GDF-15 analogues and leptin can enhance weight loss [19]. Inhibiting GDF-15 has a therapeutic effect on cachexia-related complications [130]. Genetic deletion of GDF-15 or GDF-15 monoclonal antibody (mAb) can effectively improve symptoms and survival [58, 131–135]. In recent years, GDF-15 mAb has received increasing attention: Visugromab (CTL-002), as a GDF-15 mAb, combined with the checkpoint inhibitor nivolumab (anti-PD-1) can be used to manage advanced solid tumors [136, 137]; AV-380 and ponsegromab also belong to anti-GDF-15 antibodies, prescribed for patients with non-small cell lung cancer, pancreatic cancer, colorectal cancer and metastatic colorectal cancer [138]; the mAB1 antibody can be used to improve cachexia and chemotherapy-induced anorexia, and enhance the efficacy of immunotherapy by promoting T-cell infiltration into the tumor microenvironment [58, 131] (Table 3). It should be noted that it is imperative to distinguish between anorexia and vomiting caused by physiological (e.g., pregnancy) and pathological GDF-15 (e.g., cancer cachexia) insult, so as to avoid interfering with its normal function. In the future, GDF-15 agonists and antagonists may be administered to manage weight loss and cachexia, respectively. The medicinal effects of GDF-15 against obesity, alongside concomitant harms to promote cachexia development, represent the main obstacles. The level of GDF-15 should be accurately adjusted according to the metabolic status of patients with obesity to avoid side effects such as cachexia. Future research is warranted to concentrate on tissue-specific regulatory strategies, in hopes of offsetting contradictory clinical applications.

**TABLE 3:** Clinical trials targeting the GDF-15/GFRAL axis in metabolic and oncological diseases.

Clinical trial identifier	Start year	Compound/Drug	Locations	Type	Target	Disease	Participants, n	Phase	Status	Results	References
NCT05865535	2023	AV380	USA	mAb	GDF-15	Cancer cachexia	30	Phase Ib	Recruiting	NA	NA
NCT05397171	2022	AZD8853	Canada, USA	mAb	GDF-15	Advanced solid tumors	17	Phase I/IIa	Terminated	AZD8853 was well tolerated; however, no objective responses or pharmacodynamic effects were seen and GDF-15 suppression was not sustained	[133]
NCT05199090	2022	MBL949	USA	Agonist/analog	GFRAL	Overweight, obesity	126	Phase II	Terminated	The prolonged half-life of MBL949 supports biweekly dosing in patients. MBL949 had an acceptable safety profile. The robust weight loss observed in nonclinical species did not translate to weight loss efficacy in humans	[135]
NCT05546476	2022	Ponsegromab	USA, Australia, Bulgaria, and others	mAb	GDF-15	Cancer cachexia	187	Phase II	Completed	The inhibition of GDF-15 with ponsegromab resulted in increased weight gain and overall activity level and reduced cachexia symptoms	[138]
NCT04803305	2021	Ponsegromab	USA, Canada	mAb	GDF-15	Advanced cancer, anorexia	18	Phase I	Completed	Ponsegromab was well tolerated, suppressed serum GDF-15 concentrations, and demonstrated preliminary evidence of efficacy	NA
NCT04299048	2020	Ponsegromab	USA	mAb	GDF-15	Cancer cachexia	11	Phase Ib	Completed	Ponsegromab was well tolerated	[134]
NCT04725474	2020	Visugromab (CTL-002)	Germany, Spain, Switzerland	mAb	GDF-15	Solid tumor	263	Phase I/IIa	Recruiting	Neutralizing GDF-15 can overcome resistance to immune checkpoint inhibition in cancer	[137]
NCT04068896	2019	NGM120	USA	mAb	GFRAL	Advanced solid tumors	89	Phase I/II	Completed	NA	NA
NCT03764774	2018	LY3463251	USA	Agonist/analog	GFRAL	Healthy people	118	Phase I	Terminated	The decrease in body weight is modest	[132]

Participant numbers reflect actual enrollment from *ClinicalTrials.gov*.

At last, in the field of geriatric nutrition, GDF-15 is connected to deteriorated nutritional status, increased malnutrition risk, and decreased physical function [32, 121]. Combined with the Global Leadership Initiative on Malnutrition (GLIM) criteria, GDF-15 may be used as a swift and sensitive indicator of malnutrition in older people [139, 140]. GDF-15 may be adopted as a readily available biomarker to monitor muscular diseases, in particular, age-related sarcopenia and comorbidity [13, 20, 68, 71, 73].

## Conclusion and future perspectives

This review summarizes the pleiotropic effects of GDF-15 on nutritional status regulation: GDF-15 participates in energy balance, MetS, and geriatric malnutrition through appetite suppression, muscle metabolism modulation, and iron homeostasis regulation. Although the anorectic effect mediated by its core receptor GFRAL has been established, its regulation of muscle atrophy, iron metabolism, MetS, and tissue function is clearly not limited to a single pathway, and the multidimensional mechanisms remain largely unexplored.

Based on current research limitations, future breakthroughs should focus on the following directions (1) While known to exert appetite inhibition and regulate glucose-lipid metabolism balance through its only identified specific receptor GFRAL, GDF-15’s precise neuroanatomical and information transmission processes require further elucidation. Moreover, the regulation of GDF-15 in muscle atrophy, iron metabolism, MetS, and physical function, among others, is evidently not completed through this pathway alone. Most current studies suggest a potential association with signaling pathways such as mitogen-activated protein kinase (MAPK), PI3K/Akt, STAT3, RET, and SMAD, but relevant mechanistic research is still lacking. Future considerations include that constructing ‘muscle-adipose-bone’ organoid co-culture system to simulate its hub role in multi-tissue metabolic crosstalk, applying single-cell transcriptomics combined with spatial proteomics to map the cellular localization of GDF-15 in target tissues like the brainstem and adipose tissue, using CRISPR-Cas9 to construct tissue-specific GDF-15 knockout models (e.g., muscle/liver/intestinal epithelium) to analyze local microenvironmental effects, and integrating clinical data with molecular dynamics simulation to predict metabolic response trajectories of GDF-15 pathway interventions. (2) Some studies indicate gender differences in the association between GDF-15 and diseases or nutritional status, such as a more significant correlation with nutritional status in females [32] and a stronger correlation between elevated GDF-15 concentration and reduced muscle mass in males [70], which may involve sex hormone variations or receptor expression differences. Establishing gender-stratified cohorts to validate how sex hormones (estrogen/androgen) regulate GDF-15 signaling and to develop gender-specific biomarker thresholds are expected to address this issue. (3) GDF-15 has certain predictive value for malnourished conditions such as muscle atrophy, tumor cachexia, geriatric physical function decline, iron metabolism disorders, and bone loss, but its clinical translation still requires large-scale prospective cohorts and Mendelian randomization studies to validate whether it acts as a causality or secondary marker of malnutrition [141]. Additionally, establishing age-stratified and disease-stage diagnostic thresholds through multi-center studies and integrating artificial intelligence algorithms to optimize predictive models are essential. (4) Although humanized mAb immunotherapies inhibiting GDF-15 (e.g., Visugromab/CTL-002, Ponsegromab) have attracted significant attention in recent years [136–138], their therapeutic application is limited by the short half-life and aggregation-prone nature of GDF-15. Developing stable delivery systems or small-molecule antagonists is necessary to overcome the clinical limitations of antibody drugs, while exploring synergistic therapeutic models combining GDF-15 inhibitors with anti-inflammatory agents (e.g., IL-6 receptor antagonists) and muscle protectants (e.g., myostatin antibodies) may enhance treatment efficacy. (5) Future research should also focus on the regulation of GDF-15 pathways by intervention strategies and their dynamic monitoring value in older patients with multiple comorbidities [117], developing minimally invasive/noninvasive real-time monitoring devices (such as wearable biosensors) to achieve continuous tracking of GDF-15 levels.

In summary, as a key molecule linking metabolic stress and tissue injury, GDF-15 provides novel targets for the prevention and treatment of nutrition-related diseases. Future efforts should integrate molecular research, clinical translation, and geriatric care, addressing sex differences, developing targeted therapeutics, and implementing multimodal intervention strategies to advance from biomarker discovery to precision therapy.

## Glossary

Acly: ATP-citrate lyase
Acc: Acetyl-CoA carboxylase
Akt: Protein kinase B
ALK/4/5/7: Activin receptor-like kinase/4/5/7
AMPK: AMP-activated protein kinase
AN: Anorexia nervosa
AP: Area postrema
Atrogin-1: Muscle atrophy F-box protein 1
Bax: Bcl-2-associated X protein
Bcl-2: B-cell lymphoma 2
C/EBPα: CCAAT/enhancer binding protein α
Caspase 3: Cysteine-aspartic protease 3
COPD: Chronic obstructive pulmonary disease
Cpt1a: Carnitine palmitoyltransferase 1A
CRH: Corticotropin-releasing hormone
ERK: Extracellular signal-regulated kinase
Fasn: Fatty acid synthase
FGF21: Fibroblast growth factor 21
FoxO3a: Forkhead box protein O3a
GC: Glucocorticoid
GDF-15: Growth differentiation factor 15
GFRAL: Glial cell-derived neurotrophic factor family receptor α-like protein
GLIM: Global Leadership Initiative on Malnutrition
HGS: Handgrip strength
HOMA-IR: Homeostatic Model Assessment for Insulin Resistance
HOP2: Homologous-pairing protein 2
HPA: Hypothalamic-pituitary-adrenal axis
IFN-γ: Interferon-γ
IgG Fc: Immunoglobulin G Fc region
IL-18/1β/6: Interleukin-18/1β/6
IR: Insulin resistance
IRS1: Insulin receptor substrate 1
JNK: C-Jun N-terminal Kinase
mAb: Monoclonal antibody
MAPK: Mitogen-activated protein kinase
MASH: Metabolic dysfunction-associated steatohepatitis
MetS: Metabolic syndrome
miR-1/133a/181a/499: microRNA-1/133a/181a/499
MNA-SF: Mini Nutritional Assessment-Short Form
mTOR: Mammalian target of rapamycin
MuRF-1: Muscle-specific RING finger protein 1
NEDD4L: Neural precursor cell expressed developmentally downregulated 4-like
NF-κB: Nuclear factor kappa B
NLRP3: NOD-like receptor protein 3
NPY: Neuropeptide Y
NTS: Nucleus of the solitary tract
PCOS: Polycystic ovary syndrome
Pgc1a: Peroxisome proliferator-activated receptor γ coactivator 1-α
PI3K: Phosphatidylinositol 3-kinase
PLC: Phospholipase C
POLG: Polymerase γ
POMC: Proopiomelanocortin
PPARα/PPARγ: Peroxisome proliferator-activated receptor α/γ
RET: Rearranged during transfection
ROS: Reactive oxygen species
SMAD2/3/4/7: Mothers against decapentaplegic homolog 2/3/4/7
STAT3: Signal transducer and activator of transcription 3
T2DM: Type 2 diabetes mellitus
TAK-1: TGF-β-activated kinase 1
TGF-β: Transforming growth factor-β
TNF: Tumor necrosis factor
ZAG: Zinc-α2-glycoprotein
